# Supplementary material for: Extracting quantitative dielectric properties from pump-probe spectroscopy
Source: Nat Commun. 2022 Mar 17;13:1437. doi: 10.1038/s41467-022-29112-y (PMC8931171; doi:10.1038/s41467-022-29112-y)
Supplement: Supplementary file 1 — Supplementary Information [file 41467_2022_29112_MOESM1_ESM.pdf]

Supplementary Information for
"Extracting Quantitative Dielectric Properties from Pump-Probe Spectroscopy"

Arjun Ashoka[1], Ronnie R. Tamming[2,3,4], Aswathy V. Girija[1], Hope Bretscher[1], Sachin Dev Verma[1†], Shang-Da Yang[5], Chih-Hsuan Lu[5], Justin M. Hodgkiss[3,4], David Ritchie[1], Chong Chen[1], Charles G. Smith[1], Christoph Schnedermann[1], Michael B. Price[3,4], Kai Chen[2,4,6], Akshay Rao [1 *]

[1] Cavendish Laboratory, University of Cambridge, J.J. Thomson Avenue, CB3 0HE, Cambridge, United Kingdom
[2] Robinson Research Institute, Faculty of Engineering, Victoria University of Wellington, Wellington 6012, New Zealand.
[3] School of Chemical and Physical Sciences, Victoria University of Wellington, Wellington 6012, New Zealand
[4] MacDiarmid Institute for Advanced Materials and Nanotechnology, Wellington 6012, New Zealand.
[5] Institute of Photonics Technologies, National Tsing Hua University, Hsinchu, 30013, Taiwan
[6] The Dodd-Walls Centre for Photonic and Quantum Technologies, Dunedin 9016, New Zealand
[†]Current Address: Department of Chemistry, Indian Institute of Science Education and Research Bhopal, Bhopal Bypass Road, Bhopal 462066, Madhya Pradesh, India.

*Correspondence: ar525@cam.ac.uk

SI 1 – Retrieving the Stimulated Emission Band from an Optical Constant Based Analysis

The KK analysis presented does not include the stimulated emission (SE) band. This is because the approach focuses solely on constructing the photoexcited weighed density of states in the material. Just like photoluminescence, which is also not included in this model, changes in the net transmission that result from radiation from the material rather than changes in optical constants are not explicitly captured in our analysis. In order to fully recover the stimulated emission band from this material, there exist two options depending on the magnitude of the Stokes shift:

a) If the Stokes shift is large, one can study the photobleaching-like negative spectral signatures in $\Delta\varepsilon_2$ that appear shifted from the expected ground state photobleaching feature (based on the static ε_2), just as is typically done in transient absorption spectroscopy.

b) However, if the Stokes shift is small, one can combine the methodology we provide to calculate $\Delta\varepsilon_{2\_KKR}$ that includes the SE band with the Frequency Domain Interferometry (FDI) retrieved $\Delta\varepsilon_{2\_FDI}$ which is sensitive solely to the real part of the refractive index and therefore does not pick up the SE band. The spectrum of $\Delta\varepsilon_{2\_KKR}$ - $\Delta\varepsilon_{2\_FDI}$ should contain purely the SE band.

SI 2 - Fresnel's Equations for Reflection and Transmission of a Thin Film and Wafer

The relationship between the spectral properties and the dielectric function/refractive index of the material are given by the well-known Fresnel Equations by solving Maxwell's equations with boundary conditions at the interfaces. Ignoring Fabry–Pérot resonances the thin film reflection and transmission are given by,

$$T = |(1 - r^2)t|^2$$

where

$$r = \frac{1 - \sqrt{\varepsilon}}{1 + \sqrt{\varepsilon}}$$

and

$$t = Exp(i\,\frac{\omega}{c}\,d\,\sqrt{\varepsilon})$$

r and t being the electric field complex reflection and transmission amplitudes respectively. Similar expressions including Fabry–Pérot resonances and for 2D materials can also be derived [1]. For the sake of brevity we use the definitions above for our thin film samples studied in reflection. For a GaAs wafer the expression for the top surface reflection,

$$R = |r|^2$$

is the only one that is needed as there is no transmission through the material.

We note that while we have ignored the interference based Fabry–Pérot resonances, similar expression for the transmission and reflection for a multilayer sample can be calculated in both the coherent and incoherent regimes using the transfer matrix method.[2]

SI 3 - Variational KK Fitting of $CsPbBr_3$ Data

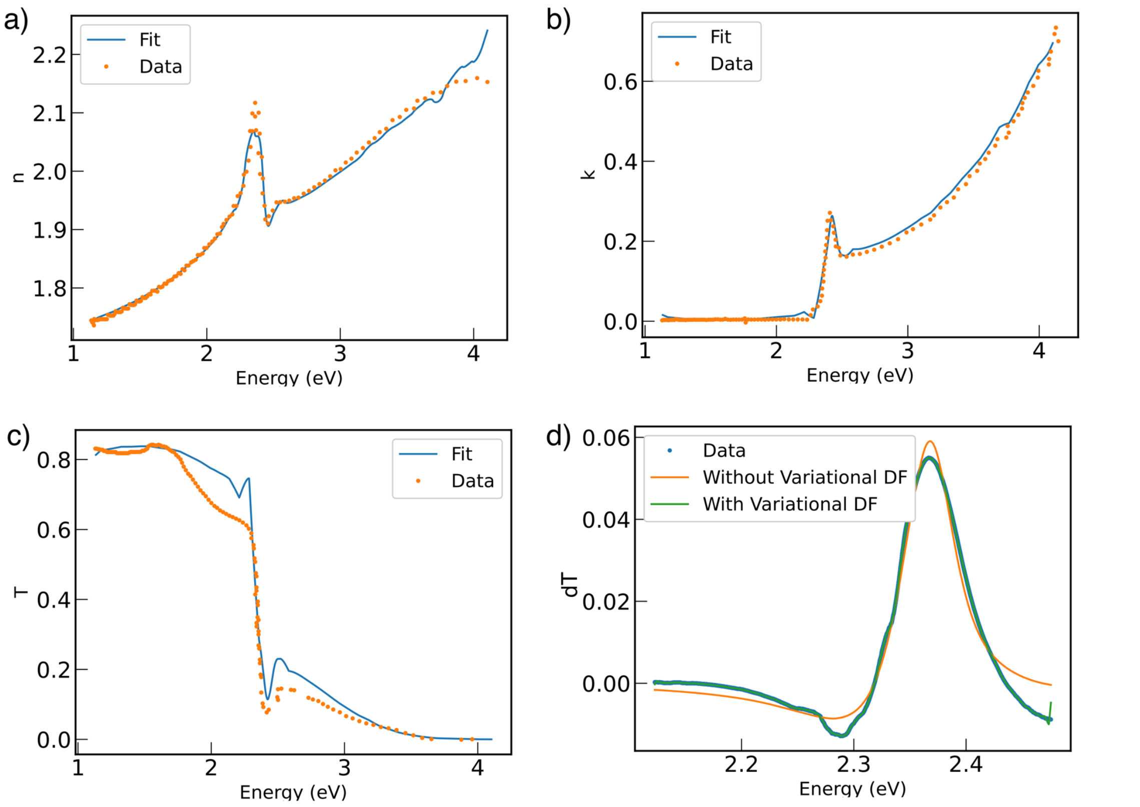

Figure S3: KK Fitting of $CsPbBr_3$. a), b) and c) are the final variational fits of the static ellipsometry and transmission data obtained from [2,3] . From the fits to this data the derivates are calculated and used to variationally fit the differential dielectric function to the transient transmission data as shown in d).

The ellipsometric and transmission data from [3,4] were taken and fit first to 3 Lorentz oscillators. These were fixed and the residues were variationally fit the triangular oscillators reported in [5]. The final fits are shown in Fig S3 a), b) and c). These fits were used to calculate the derivatives in Eqn (4) of the main text. The differential transmission at a given time delay was then fit using the differential dielectric function expansion and the final fit including the variational oscillators are shown in Fig S3 d). The last step was repeated for all time points.

SI 4 – Contribution of dn to the transient transmission in $CsPbBr_3$ at 500 fs

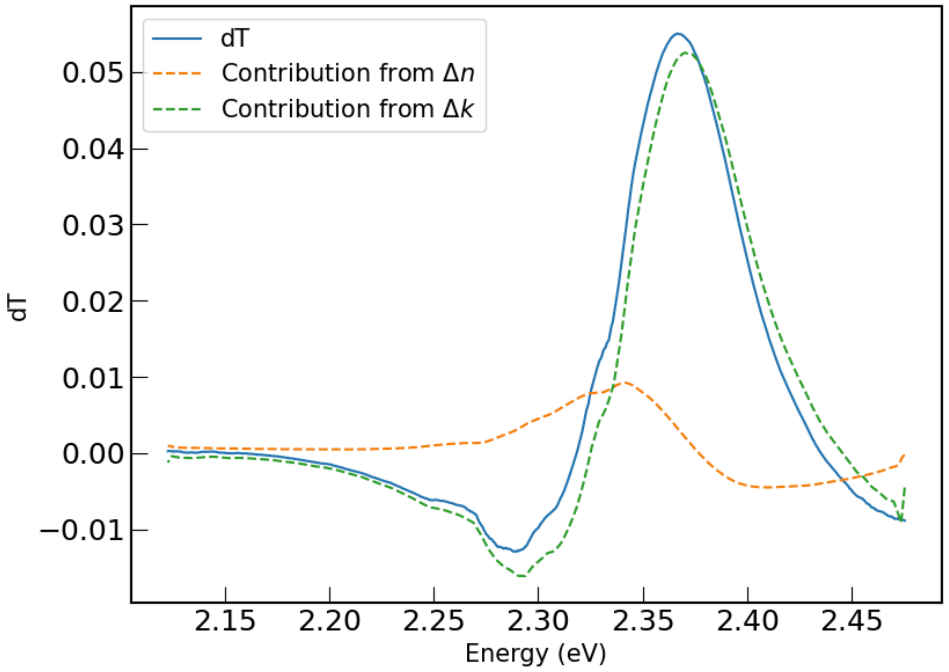

Figure S4: Contribution of dn and dk to the differential transmission of $CsPbBr_3$ at 500fs demonstrating that the sub-gap transient transmission can be partially explained by the refractive index change.

SI 5 - Variational KK Fitting of Pentacene

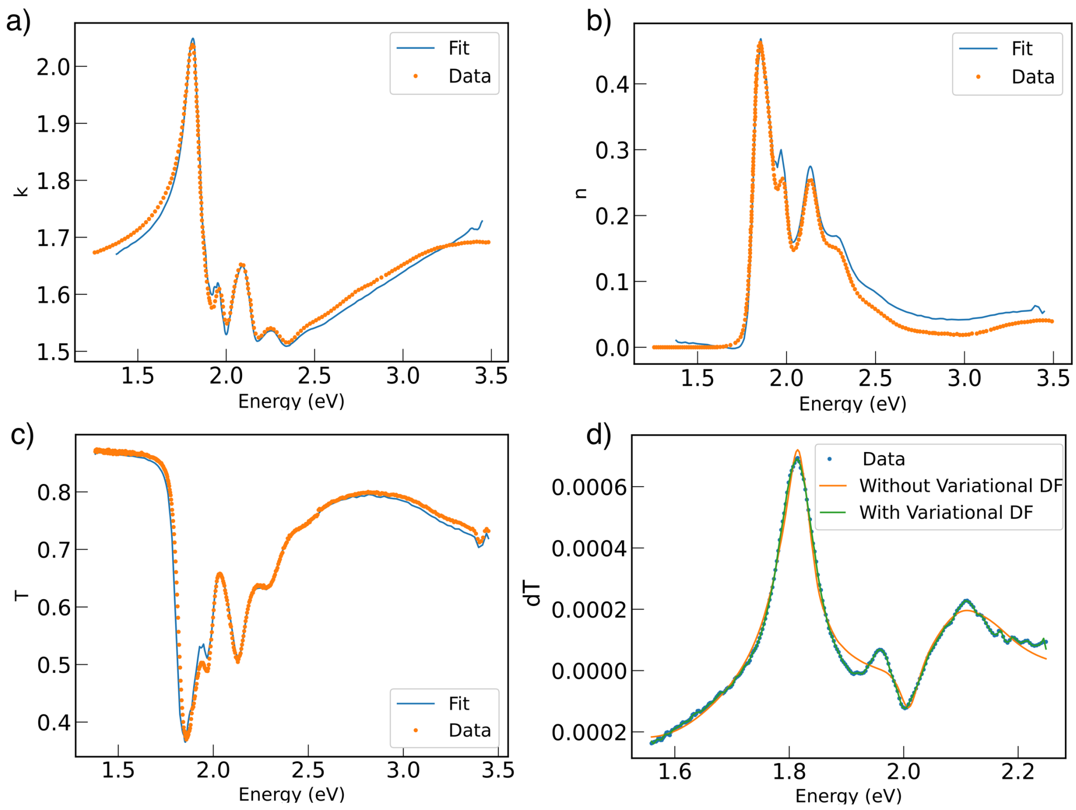

Figure S5: KK Fitting of Pentacene. a), b) and c) are the final variational fits of the static ellipsometry and transmission data obtained from [5]. From the fits to this data the derivates are calculated and used to variationally fit the differential dielectric function to the transient transmission data as shown in d).

The ellipsometric data from [6] and measured transmission were taken and fit first to 5 Lorentz oscillators. These were fixed and the residues were variationally fit to the triangular oscillators reported in [5]. The final fits are shown in Fig S5 a), b) and c). These fits were used to calculate the derivatives in Eqn (4) of the main text. The differential transmission at a given time delay was then fit using the differential dielectric function expansion and the final fit including the variational oscillators are shown in Fig S5 d). The last step was repeated for all time points.

<u>SI 6 – Variational KK Fitting of GaAs</u>

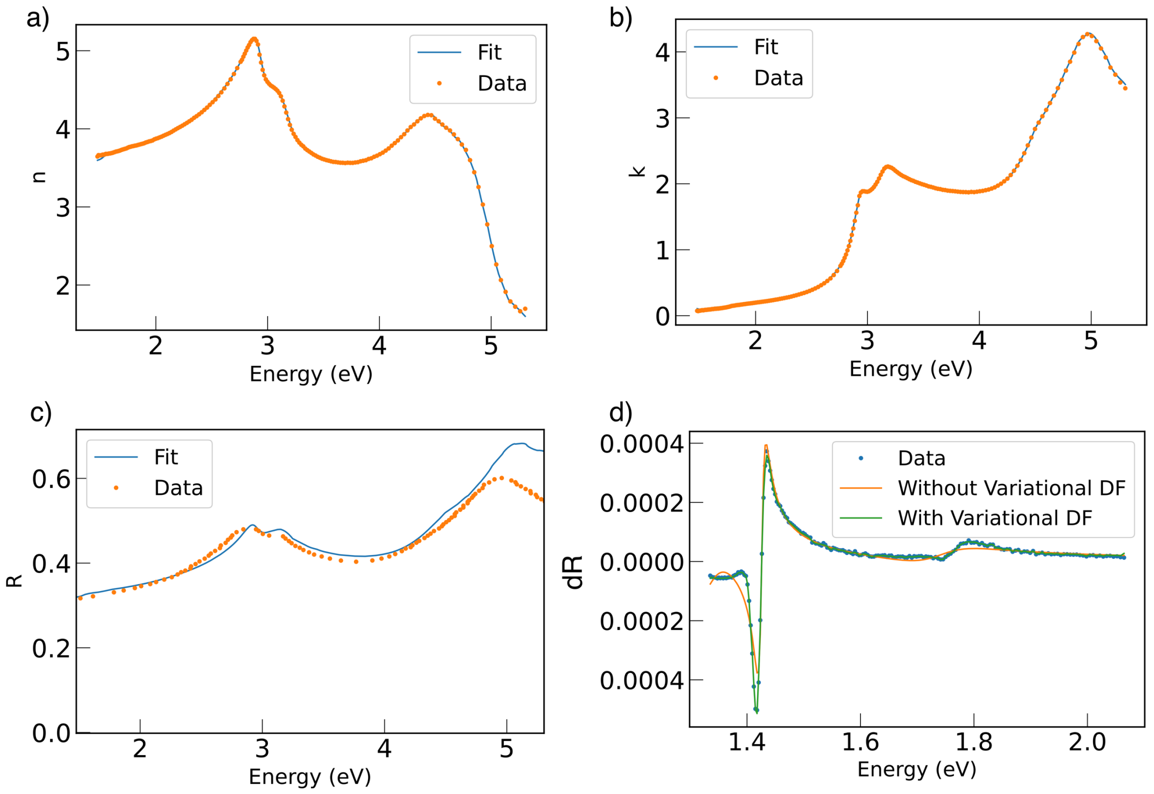

Figure S6: KK Fitting of GaAs. a) and b) are the final variational fits of the static ellipsometry and c) is the recovered reflectivity data obtained from [6] compared to that from [7]. From the fits to this data the derivates are calculated and used to variationally fit the differential dielectric function to the transient reflection data as shown in d).

The ellipsometric data from [7] was taken and fit first to 5 Lorentz oscillators. These were fixed and the residues were variationally fit the triangular oscillators reported in [5]. The final fits are shown in Fig S6 a) and b). c) shows the retrieved reflection spectrum compared to that measured by [8]. These fits were used to calculate the derivatives in Eqn (4) of the main text except here we calculate the differential reflectance instead of the transmission. These are used to then fit the measured differential reflectance at a given time delay using the differential dielectric function expansion and the final fit including the variational oscillators are shown in Fig S6 d). The last step was repeated for all time points.

1. Sie, E. J., Frenzel, A. J., Lee, Y. H., Kong, J. & Gedik, N. Intervalley biexcitons and many-body effects in monolayer MoS2. *Phys. Rev. B - Condens. Matter Mater. Phys.* **92**, 1–8 (2015).
2. Troparevsky, M. C., Sabau, A. S., Lupini, A. R. & Zhang, Z. Transfer-matrix formalism for the calculation of optical response in multilayer systems: from coherent to incoherent interference. *Opt. Express* **18**, 24715 (2010).
3. Yan, W. *et al.* Determination of complex optical constants and photovoltaic device design of all-inorganic CsPbBr 3 perovskite thin films . *Opt. Express* **28**, 15706 (2020).
4. Maqbool, M. *et al.* Structural, electronic and optical properties of CsPbX3(X=Cl, Br, I) for energy storage and hybrid solar cell applications. *J. Alloys Compd.* **705**, 828–839 (2017).
5. Kuzmenko, A. B. Kramers-Kronig constrained variational analysis of optical spectra. *Rev. Sci. Instrum.* **76**, 1–9 (2005).
6. Hinderhofer, A. *et al.* Optical properties of pentacene and perfluoropentacene thin films. *J. Chem. Phys.* **127**, 1–6 (2007).
7. Aspnes, D. E., Kelso, S. M., Logan, R. A. & Bhat, R. Optical properties of AlxGa1−x As. *J. Appl. Phys.* **60**, 754–767 (1986).
8. Alonso, M. I. & Garriga, M. Optical properties of semiconductors. *Springer Ser. Opt. Sci.* **212**, 89–113 (2018).
